# A unified approach to false discovery rate estimation

**DOI:** 10.1186/1471-2105-9-303

**Published:** 2008-07-09

**Authors:** Korbinian Strimmer

**Affiliations:** 1 Institute for Medical Informatics, Statistics and Epidemiology, University of Leipzig, Härtelstr. 16-18, 04107 Leipzig, Germany

## Abstract

**Background:**

False discovery rate (FDR) methods play an important role in analyzing high-dimensional data. There are two types of FDR, tail area-based FDR and local FDR, as well as numerous statistical algorithms for estimating or controlling FDR. These differ in terms of underlying test statistics and procedures employed for statistical learning.

**Results:**

A unifying algorithm for simultaneous estimation of both local FDR and tail area-based FDR is presented that can be applied to a diverse range of test statistics, including *p*-values, correlations, *z*- and *t*-scores. This approach is semipararametric and is based on a modified Grenander density estimator. For test statistics other than *p*-values it allows for empirical null modeling, so that dependencies among tests can be taken into account. The inference of the underlying model employs truncated maximum-likelihood estimation, with the cut-off point chosen according to the false non-discovery rate.

**Conclusion:**

The proposed procedure generalizes a number of more specialized algorithms and thus offers a common framework for FDR estimation consistent across test statistics and types of FDR. In comparative study the unified approach performs on par with the best competing yet more specialized alternatives. The algorithm is implemented in R in the "fdrtool" package, available under the GNU GPL from  and from the R package archive CRAN.

## Background

The false discovery rate (FDR) plays a prominent role in many high-dimensional testing and model selection procedures. Consequently, FDR methodologies are ubiquitous in the analysis of high-throughput data, such as in differential gene expression, SNP biomarker selection, peak detection in proteomic mass spectrometry data, or inference of edges in a network.

False discovery rate analysis starts with the seminal works by Schweder and Spjøtvoll [1] and by Benjamini and Hochberg [2]. Many others have followed suite, so that to date an impressive number of different algorithms for controlling and estimating false discovery rates have appeared in the literature.

In a nutshell, FDR estimation algorithms may be broadly categorized by the type of

• FDR,

• input test statistic, and

• employed inference procedures.

There are two main types of FDR, the "classic" tail area-based FDR (= Fdr) and local FDR (= fdr). Most FDR procedures are concerned either with Fdr or fdr, simultaneous estimation of both types of FDR is only possible with a few selected algorithms. With regard to test statistics, FDR calculations typically rely on *p*-values. However, FDR can be easily extended to other test statistics, such as correlations [3]. Relaxing the requirement of having *p*-values as input has the additional benefit that it allows for empirical null modeling [4]. Further key differences among the various FDR methods relate primarily to their respective procedures for density estimation and for inferring the proportion of null statistics.

Here, a unified statistical procedure for FDR estimation is described that generalizes a number of previous algorithms, specifically those of [5,6,4] and [7]. Notable features of thus approach include simultaneous estimation of Fdr and fdr from a diverse range of test statistics, its simplicity, very little a prior modeling assumptions, and the option of fitting the empirical null model.

The remainder of this paper is set out as follows. In the first part of the 'Methods' section a brief overview is given of the basic theory and definitions related to FDR and its estimation. In the second part of the 'Methods' section the proposed unified FDR procedure is described in detail. In the remaining part of the paper the new procedure is evaluated in comparison with other competing FDR estimation schemes.

## Methods

### Basic theory of FDR

This section gives a very brief review of the two component FDR model and the local and tail area-based FDR criteria. For a more refined discussion it is referred to [8] and references therein.

Throughout the paper the Efron naming conventions are followed. Specifically, "fdr" denotes the local false discovery rate, "Fdr" denotes the tail area-based false discovery rate, and "FDR" is a generic term encompassing both variants. Similarly, FNDR is the generic abbreviation for the false non-discovery rate [9].

In the following *m *simultaneous tests are considered, resulting in *m *test statistics such as *t*_1_,...,*t*_*m *_or *z*_1_,...,*z*_*m *_and corresponding *p*-values *p*_1_,...,*p*_*m*_.

#### Tail area-based FDR

In order to control the number of false discoveries, i.e. the expected ratio *E*(*V/R*) of the number of false positives *V *among all significant tests *R*, Benjamini and Hochberg [2] introduced the following linear step-up procedure. First, the *p*-values are ordered so that *p*_(1) _≤ ... ≤ *p*_(*m*)_. Second, each value *p*_(*i*) _is compared with $q\frac{i}{m}$, where *q *is the desired FDR level. Finally, with *k *= max(*i *: *p*_(*i*) _≤ $q\frac{i}{m}$) all hypotheses belonging to *p*_(1)_,...,*p*_(*k*) _are rejected. [2] show that when the test statistics are independent then this procedure controls *E*(*V/R*) at level ≤ *q*.

The above procedure suggests the following simple correction of *p*-values, in the following called Benjamini-Hochberg (BH) rule:

(1) $$\begin{array}{cc} p_{i}^{\text{BH}}=p_{i}\frac{m}{\text{order}(p_{i})}, & i=1,...,m \end{array}.$$

Here order(*p*_*i*_) equals one for the smallest and *m *for the largest *p*-value, respectively. For comparison, the standard Bonferroni correction [10] is $p_{i}^{\text{Bf}}=p_{i}m$, and hence $p_{i}\leq p_{i}^{\text{BH}}\leq p_{i}^{\text{Bf}}$.

A way to intuitively understand BH rule is to consider the following two-component mixture of the observed *p*-values,

(2) $$\begin{array}{c} f(p)=\eta_{0}f_{0}(p)+(1-\eta_{0})f_{A}(p)\\ =\eta_{0}+(1-\eta_{0})f_{A}(p). \end{array}$$

For *p*-values the null density *f*_0 _is the uniform distribution U(0,1) and corresponds to the "uninteresting" *p*-values, whereas *f*_*A *_is an unspecified alternative density for the "interesting" *p*-values. This mixture model may also be written in terms of distribution functions,

(3) $$\begin{array}{c} f(p)=\eta_{0}F_{0}(p)+(1-\eta_{0})F_{A}(p)\\ =\eta_{0}p+(1-\eta_{0})F_{A}(p). \end{array}$$

**Figure 1:**
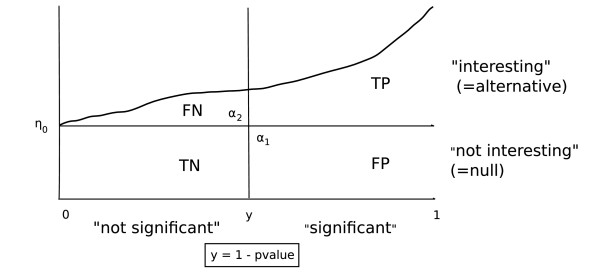
**Two-component mixture model for *p*-values with cutoff point *y*_*c*_. This implies a decision rule with errors *α*_1 _and *α*_2_.** Further abbreviations: FP, false positives; TP, true positives; FN, false negatives; TN, true negatives. Note that here these quantities are all fractions (not counts) and that FP + TP + FN + TN = 1.

Fig. 1 illustrates the *p*-value mixture model using the transformed statistic *y *= 1 - *p*.

This two-component model provides the means for *defining *the tail area-based false discovery rate "Fdr"and also the false non-discovery rate "Fndr"[9]. Specifically, Fdr(*p*) = *η*_0_*p*/*F*(*p*) – see also Table 1. This "Bayesian" definition of "Fdr" [11] is closely related but not identical to the original approach by Benjamini and Hochberg. The key difference is that being density-based it implicitly assumes that the number of hypotheses is large (*m *→ ∞). Intriguingly, this allows to view most FDR procedures based on the observed test statistics as providing *estimates *of "Fdr" (note the subtle but important difference of estimating FDR versus controlling FDR).

**Table 1:** Definitions of FDR quantities contrasted with that of specificity and sensitivity.

*Quantity*	*Definition*	
Fdr	= Prob("not interesting"|*Y *= *y*)	$=\frac{FP}{TP+FP}$
Fndr	= Prob("interesting"|*Y *= *y*)	$=\frac{FN}{FN+TN}$

Sensitivity, Power	= Prob(*Y *= *y*|"interesting")	$=\frac{TP}{TP+FN}$
Specificity	= Prob(*Y *= *y*|"not interesting")	$=\frac{TN}{TN+FP}$

fdr	= Prob("not interesting"|*Y *= *y*)	
fndr	= Prob("interesting" *|Y *= *y*)	= 1 - fdr

Note that in the above definitions TP, FP, FN, FP are assumed to be the true fractions – see Fig. 1. If instead observed counts are used, additionally the expectation *E*(.) must be taken, conditioned on seeing a non-zero denominator (cf. [7,11]).

In case of the BH-corrected *p*-values (Eq. 1), it turns out that this rule is simply the nonparametric empirical estimator of Fdr:

$$\begin{array}{c} \text{fdr}(p_{i}):=\text{Prob}("\text{not interesting}"|P\leq p_{i})\\ =\frac{\eta_{0}F_{0}(p_{i})}{F(p_{i})}=\frac{\eta_{0}p_{i}}{F(p_{i})}. \end{array}$$

Plugging in the empirical cumulative density function (ECDF) $\hat{F}(p_{i})=\frac{\text{order}(p_{i})}{m}$ as estimator of *F*(*p*) and using the conservative guess ${\hat{\eta }}_{0}$ = 1 yields

$$\begin{array}{c} \hat{\text{Fdr}}(p_{i})=\frac{{\hat{\eta }}_{0}p_{i}}{\hat{F}(p_{i})}=p_{i}\frac{{\hat{\eta }}_{0}m}{\text{order}(p_{i})}\\ \leq p_{i}\frac{m}{\text{order}(p_{i})}. \end{array}$$

It is instructive to compare the definitions of "Fdr" and "Fndr" for a given threshold *y *with those of "sensitivity" and "specificity" – see Table 1. Note that the order of conditioning is reversed in the two instances, but otherwise the definitions are very similar. Furthermore, both "Fdr-Fndr" and "sensitivity-specificity" offer the means for determining an optimal decision rule. In a conventional test situation the threshold *y *is chosen to maximize both sensitivity and specificity (i.e. typically specificity is fixed and power is maximized). Analogously, in an FDR analysis one seeks to minimize Fdr and Fndr (e.g, by fixing Fndr and minimizing Fdr). Hence, there is a tradeoff between Fndr and Fdr, just as there is a tradeoff between sensitivity and specificity. Note that the formal similarities between Fdr/Fndr and sensitivity/specificity is yet another reason for prefering the Bayesian variant of FDR over other more operational definitions.

The BH rule is popular due to its simplicity. However, often it is a rather conservative estimator of Fdr. One way to improve the BH rule is to substitute a more appropriate estimate of the null proportion *η*_0_. This leads directly to the well-known *q*-values, which are refined BH estimates with various suggested options for the estimation of *η*_0 _[12,7].

Additionally, monotonicity is another issue where the BH rule is open to improvement. Specifically, direct application of the BH correction easily yields corrected *p*-values with a different ordering than that of the original test statistics. This unpleasant property has already been noted by [2], and indeed the "max" function in the original step-up procedure provides a corresponding fix (albeit a rather adhoc one). [5,6] point out that this issue can be more elegantly resolved by requiring the distribution function *F*(*p*) of the *p*-values to be concave and, correspondingly, the marginal density *f*(*p*) to be monotonically decreasing. There many different ways for fitting the two component FDR mixture model (Eqs. 2 and 3) and for estimating densities and *η*_0_. This explains the multitude of FDR approaches in existence. Common to all is some form of "zero assumption" to render the mixture model identifiable. Typically, for large *p*-values one assumes that there is no contamination with the alternative distribution, i.e. Fndr(*p *→ 1) = 0 and therefore *f*(*p *→ 1) = *η*_0_

#### Local FDR

An alternative to the classic tail-area based FDR is the local FDR, abbreviated here as "fdr". Specifically, the local FDR is the probability of the null model conditioned the observed test statistic (see Table 1). Note that the local FDR takes is computed at the density level, in contrast to the Fdr that is based on cumulative densities.

This approach has mainly been advocated by Efron and a few others [13-15]. The key virtue of local FDR is that it is more readily interpretable than Fdr, as it is an empirical Bayesian posterior probability and not some variant of a corrected *p*-value. However, due to the use of densities it is also more difficult to estimate, in particular if the alternative distribution in the two-component model is not parametrically specified.

An important relation between Fdr and fdr is the property Fdr(*p*) ≤ fdr(*p*) that holds if fdr(*p*) is monotonically decreasing with decreasing *p*-value.

#### Test statistics other than *p*-values and empirical null modeling

Virtually all FDR procedures – both local and tail area-based methods – are designed to work with *p*-values as input test statistics. Regardless the popularity of *p*-value-based approaches, in many instances it is often more beneficial to base the FDR calculations on the actual test statistic, such as on a regularized *t*-score, a *z*-score, or a correlation, rather than on a *p*-value.

The reason for this is as follows. Very often the theoretical null model is misspecified, due to dependencies among test statistics and other factors [16]. In turn, this may lead to overly pessimistic or too optimistic *p*-values, and thus to a violation of the implicit assumption of the FDR two-component model for *p*-values (namely that the null *p*-values are drawn from the uniform distribution). In such a case the resulting FDR values will also be biased, and thus unreliable.

Efron has shown that this can be elegantly avoided by retaining free parameters in the null model for the original test statistics (typically for location or scale) and estimating these parameters from the data [4]. Intriguingly, this *empirical null modeling *is greatly facilitated by high dimensions – and hence it is one of the few instances where high-dimensionality is not a curse but a blessing. There are various attempts to take account of the dependencies among *p*-values in FDR calculations, however it seems much more natural (and easier) to simply conduct Fdr and fdr calculations on the level of the original test statistic whilst employing an empirical null. In a recent paper these considerations are confirmed from a decision theoretic point of view [17].

Despite these apparent advantages empirical null modeling is currently available in only two FDR estimation algorithms, "locfdr" [4] and "fdrtool" (this paper). Note that fitting an empirical null is not tied to *z*-scores and the assumption of a normal null distribution, it is equally well feasible for any other test statistic, e.g., correlations [3].

### Unified procedure for FDR estimation

#### Overview and motivation

From the discussion in the previous section it is clear that there exists a veritable range of FDR-related methods. An brief overview is given in Table 2, which lists thirteen FDR procedures for which an implementation for the R platform [18] is available.

**Table 2:** Overview over some commonly used FDR estimation procedures.

*Name*	*FDR Type*	*Input Data*	*Comments*	*Description of Algorithm*
fdrtool	fdr, Fdr	*p*-values,	Modified Grenander density,	This paper
		*z*-scores,	estimate, empirical null model,	
		*t*-scores, correlations.	selection of truncation point by FNDR.	
BUM	fdr, Fdr	*p*-values	Completely parametric model.	[27]
SAGx	fdr, Fdr	*p*-values	Grenander density estimate.	[5,6]

qvalue	Fdr	*p*-values	Diverse estimates of *η*_0 _available.	[12,7]
nFDR	Fdr	*p*-values	Bernstein polynomial density.	[24]
multtest	Fdr	*p*-values	Benjamini-Hochberg algorithm.	[2]
LBE	Fdr	*p*-values	Location-based estimator.	[32]

locfdr	fdr	*z*-scores	Regression spline density estimate, empirical null model.	[4,28]
nomi	fdr	*z*-values	Normal mixture modeling of non-null density.	[22]
LocalFDR	fdr	*p*-values	Uses loess smoothing.	[15]
kerfdr	fdr	*p*-values	Kernel density estimator.	[23]
twilight	fdr	*p*-values	KS fit of truncation point.	[30]
localFDR	fdr	*p*-values	Based on stochastic order model.	[33]

Note: The first column refers to an R package or R script implementing the respective FDR algorithm.

The aim of this paper is to introduce an FDR estimation procedure that brings together many aspects that otherwise are only considered separately into one common and coherent setting. Thus, in a sense this offers a unified algorithm for FDR analysis.

Specifically, a procedure is proposed

• for the simultaneous estimation of both Fdr and fdr, regardless of the type of test statistic,

• that does not treat *p*-values any different from other test statistics,

• that maintains the ordering of original test statistics,

• that uses efficient and well tested techniques for estimating *η*_0 _and null distribution,

• and that remains (largely) compatible with the well established "locfdr" and "qvalue" algorithms.

Furthermore, the algorithm is conceptually simple. Components in this scheme for Fdr/fdr analysis are a generalized definition of the test statistic, a non-parametric density estimator, an approach of fitting the null model, combined together in an effective fashion.

The present approach, discussed in detail in the following subsections, is best described as a marriage of the non-parametric Grenander approach of [5] and [6] with the empirical null modeling of [4]. An implementation is available in the R package "fdrtool" [19].

#### Generalized test statistic

Central to the algorithm is a generic definition of the underlying test statistic. Specifically, a statistic *y *≥ 0 is considered with properties such that large values of *y *indicate an "interesting" case, and, conversely, small values close to zero an "uninteresting" case. Examples for suitable statistics *y *include:

• *y *= 1 - *p *where *p *is a *p*-value,

• *y *= |*z*| where *z *is a normal score,

• *y *= |*r*| where *r *is a correlation, and

• *y *= |*t*| where *t *is a *t*-score.

Note that the choice of test statistic *y *automatically implies a corresponding null model *f*_0_(*y*; *θ*), e.g., the uniform, half-normal, etc., which possibly contains some parameters *θ*. In terms of *y *the two-component model becomes

(4) *f*(*y*) = *η*_0_*f*_0_(*y*; *θ*) + (1 - *η*_0_)*f*_*A*_(*y*)

and

(5) *F*(*y*) = *η*_0_*F*_0_(*y*; *θ*) + (1 - *η*_0_)*F*_*A*_(*y*).

Accordingly, for a test statistic *y *the local FDR and the tail area-based FDR are given by

(6) $$\text{fdr}(y)=\eta_{0}\frac{f_{0}(y;\theta )}{f(y)}$$

and

(7) $$\text{Fdr}(y)=\eta_{0}\frac{1-F_{0}(y;\theta )}{1-F(y)}.$$

Furthermore, the *p*-value corresponding to the test statistic *y *equals 1 - *F*_0_(*y*; *θ*).

#### Density estimation using a modified Grenander approach

A central part of FDR analysis consists of the estimation of the marginal density *f*(*p*) and the associated distribution function *F*(*p*) from *p*-values *p*_*i*_corresponding to the observed test statistics *y*_*i*_.

The most simple approach is to use the empirical cumulative density function (ECDF) as estimator of *F*(*p*). Note that the ECDF is the non-parametric maximum-likelihood estimate (NPMLE). The ECDF is very widely used in FDR analysis, including the two most popular FDR approaches (BH rule, "qvalue" algorithm). However, the ECDF has the disadvantage that it requires careful post-processing in order to achive monotone FDR values. Furthermore, it is a non-trivial issue to derive a density from the ECDF (see, e.g., [15] for an approach using loess smoothing). This important if computation of local FDR values is desired.

Another popular option, pursued in the "locfdr" program, is to estimate the density by spline Poisson regression on the histogram counts [21]. This work extremely well in general but can be problematic if the distribution has a strong peak – which is not uncommon, e.g., for *p*-values or partial correlations. Furthermore, this approach introduces additional parameters such as the histogram bin width or the degrees of freedom of the spline, which for some data may need diligent adjustment. In addition, as the approach does not place any monotonicity constraints on the density there is no guarantee that the order of the scores is maintained in the corresponding FDR values.

Other possibilities recently proposed for FDR density estimation include, e.g., normal mixtures [22], kernel-based approaches [23] and Bernstein polynomials [24].

An further alternative approach is provided by the Grenander density estimator [25]. In contrast to most other density estimators it has two main benefits which are highly useful in the context of FDR estimation: it explicitly incorporates monotonicity constraints (to preserve ordering of original test statistics) and provides simultaneous estimates of both PDF and CDF (to allow computation of both fdr and Fdr). Nonetheless, it is only slightly more complicated than than the ECDF. For FDR analysis the Grenander estimator has been first suggested by [5] and by [6].

**Figure 2:**
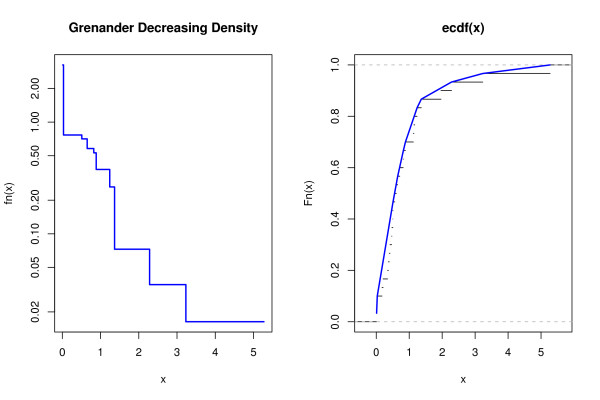
**Illustration of the Grenander density estimator.** Left: Grenander density estimate (blue line); right: the corresponding concave distribution function (blue line) and the underlying ECDF (thine black lines).

Fig. 2 illustrates the mechanics behind Grenander density estimate. In essence, the Grenander density estimator is the decreasing piecewise-constant function equal to the slopes of the least concave majorant (LCM) of the ECDF. In the example shown in Fig. 2 the data *x *are *n *= 30 random samples from the exponential distribution with mean one. The left part of the figure shows the estimated monotonically decreasing density and the right part the corresponding empirical cumulative distribution. Note that the resulting distribution *F *is piecewise linear, whereas the density *f *is piecewise constant. The Grenander estimator is easy to obtain, as the LCM of the ECDF can be computed by monotone regression with weights [26]. Specifically, let *x*_*i *_and *y*_*i *_denote the coordinates for the ECDF, and Δ*x*_*i *_= *x*_*i*+1 _- *x*_*i *_and Δ*y*_*i*_= *y*_*i*+1 _- *y*_*i*_. The slopes of the LCM are then given by antitonic regression of the raw slopes Δ*x*_*i*_/Δ*y*_*i *_with weight Δ*x*_*i *_(see also the corresponding functions monoreg, lcmgcm and grenander in the "fdrtool" package). Like the ECDF, the Grenander estimator is also the NPMLE, with the added constraint of an underlying decreasing density.

Unfortunately, the standard Grenander estimator exhibits a severe shortcoming when applied the two-component FDR model: it leads to inconsistencies with the estimated *η*_0_. This problem is extensively discussed in [5], and in fact causes these authors to abandon the Grenander estimator despite its favorable properties.

The point that is made here is that this deficiency can be easily fixed. Specifically, it is argued that the Grenander estimator is indeed very well suited for FDR analysis, but needs further modification in order to satisfy the additional constraints imposed by the two-component model.

The key problem can be understood best by going back to Eq. 3 which describes the FDR *p*-value mixture model on the level of the CDF. Effectively, this equation implies two constraints that any distribution compatible with the two-component model must satisfy:

• First, the CDF has to fulfill the condition *F*(*p*) ≥ *η*_0_*p *because *F*(*p*) = *η*_0_*p *+ (1 - *η*_0_)*F*_*A*_(*p*).

• Second, the condition 1 - *F*(*p*) ≥ *η*_0_(1 - *p*) must be met, because of

1 - *F*(*p*) = *η*_0_(1 - *p*) + (1 - *η*_0_)(1 - *F*_*A*_(*p*)).

**Figure 3:**
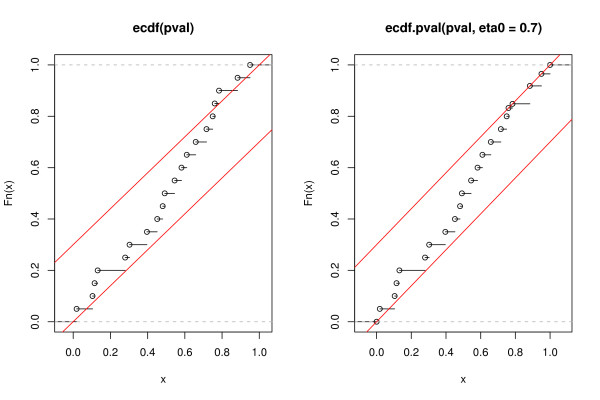
**Constraints imposed by the *p*-value mixture model on the ECDF of *p*-values.** The lower diagonal line corresponds to the constraint *F*(*p*) ≥ *η*_0_*p *whereas the upper diagonal line represents the constraint 1 - *F*(*p*) ≥ *η*_0_(1 - *p*). Right: the unmodified ECDF; left: the ECDF subject to the constraint *η*_0 _= 0.7.

The second constraint is easy to overlook but is particularly important as Fig. 3 illustrates. There, it can be seen that the two-component model enforces a corridor of allowed values of the ECDF, where the width of this corridor depends on the parameter *η*_0_. Note that the upper boundary (second constraint) ensures that the *minimum *possible slope equals *η*_0_.

For FDR calculation this has the following consequence. The ECDF need not only be modified for monotonicity (Grenander estimator) but also need to be tailored to fit the constraints of the two-component model. This can be done as follows:

1. Compute the ECDF on the basis of the *p*-values.

2. Given *η*_0 _impose the mixture model conditions on the ECDF for the *p*-values. Specifically, set ${\hat{F}}^{\prime }$(*p*_*i*_) = *η*_0_*p*_*i *_if $\hat{F}$(*p*_*i*_) <*η*_0_*p*_*i *_(i.e. obey the lower boundary shown in Fig. 3) and likewise set ${\hat{F}}^{\prime }$(*p*_*i*_) = 1 - *η*_0 _(1 - *p*_*i*_) if $\hat{F}$(*p*_*i*_) > 1 - *η*_0 _(1 - *p*_*i*_) (upper boundary).

3. The "modified" Grenander estimator is obtained as the standard Grenander estimator computed from the modified ECDF.

**Figure 4:**
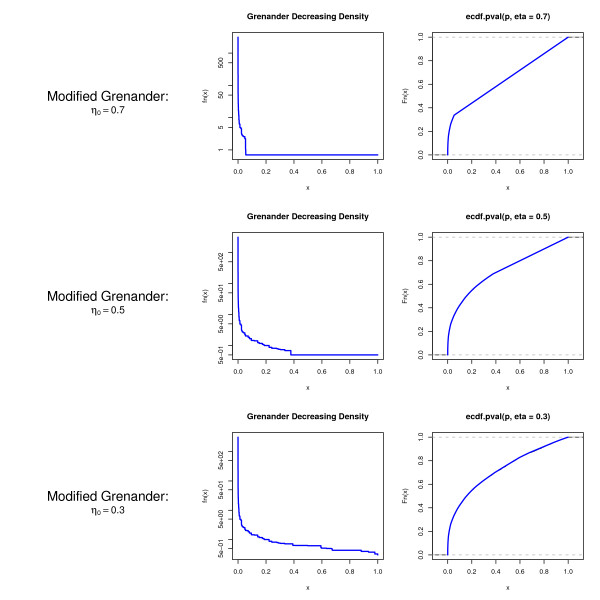
**The modified Grenander estimator computed from *p*-values for three different choices of *η*_0_.** Note that the underlying data are the same in all three instances and that the density of the modified Grenander equals *η*_0 _for large *p*-values.

Note that the modified Grenander estimator retains the key property of the standard Grenander estimator (i.e. monotonicity) but in addition satisfies the constraints imposed by the two-component mixture model model. In particular, there are no inconsistencies with respect to the parameter *η*_0_. This is illustrated in Fig. 4 where the modified Grenander estimator is applied with three different settings of *η*_0 _to an example *p*-value data set. Note that by construction the modified Grenander density equals *η*_0 _for large *p*-values.

#### Estimation of null sub-density by truncated maximum-likelihood

For computing *p*-values and the modified Grenander density suitable estimates of the parameters *θ *and *η*_0 _are required. In other words, the null sub-density *η*_0_*f*_0_(*y*; *θ*) of the two-component model (Eqs. 4 and 5) needs to be fit to the observed test statistics. This is straightforward in fully parametric models such as BUM [27]. However, it is often preferred to leave *f*_*A*_(*y*) unspecified. As a consequence, standard procedures for inferring mixture models such as the EM algorithm cannot be applied.

Instead, a truncated maximum-likelihood approach is applied here. In more detail, in this method the data are *censored *at some threshold *y*_*c*_, so that only test statistics **y**_*t *_= {*y*_*i *_: *y*_*i *_<*y*_*c*_} are retained. The underlying assumptions is that for *y*_*i *_<*y*_*c *_(nearly) all data points belong to the null part. This is called the "strong zero assumption" in [28]. The truncated null density becomes $f_{0}^{t}$(*y*; *θ*) = *f*_0_(*y*; *θ*)/*F *(*y*_*c*_; *θ*) for *y < y*_*c *_and $f_{0}^{t}$ = 0 otherwise. Maximization of the corresponding likelihood function returns ${\hat{\eta }}_{0}$ as well as an estimate of its asymptotic error. Similarly, the proportion of null values *η*_0 _is inferred by assuming a binomial model for the observed number *m*_*t *_of hypotheses in the set **y**_*t*_, which leads to the simple estimate ${\hat{\eta }}_{0}=\max\{1,\frac{m_{t}}{m}/F(y_{c};\hat{\theta })\}$ plus an associated error.

Truncated maximum-likelihood is the basis of the "locfdr" MLE algorithm [29,28]. If the test statistics are *p*-values then the truncated maximum-likelihood algorithm reduces to the simple cutoff technique used in "qvalue" and most other *p*-value-based packages.

#### Selection of suitable truncation point using the false non-discovery rate

Fitting the null model and the associated parameters *θ *and *η*_0 _by truncated maximum-likelihood depends on the choice of a suitable cutoff point *y*_*c*_. In general, one wishes to select *y*_*c *_such that the threshold is small enough to ensure that the zero assumption is met and that there is minimal bias due to contamination with the alternative *f*_*A*_(*y*). On the other hand, *y*_*c *_needs be chosen large enough so that the number of data points in **y**_*t *_is sufficient for reliably estimating *θ *and *η*_0_.

The default "smoothing" approach employed in "qvalue" specifically aims at achieving an unbiased estimate of *η*_0 _[7]. This is obtained by varying the cutoff point between zero and one, and subsequently estimating *η*_0 _by interpolation at *y*_*c *_= 1, i.e. for complete censoring.

For empirical null modeling the choice of an optimal *y*_*c *_is more complicated. Table 3 lists the algorithms employed in various versions of the "locfdr" algorithm. Essentially, "locfdr" either uses a fixed *y*_*c *_or it relies on a heuristic analytical formula aimed at reducing the mean squared error of the null sub-density [28]. Both approaches are not straightforward to extend to arbitrary test statistics *y*_*i*_. Instead, here a more simple alternative procedure is proposed that enforces the "zero assumption" by requiring that the false non-discovery rate (Fndr) is minimized (i.e. *y*_*c *_is chosen such that Fndr(*y*_*c*_) is small). Intriguingly, this leads to the following circular inferential problem: in order to determine a suitable cutoff *y*_*c *_the Fndr must be known, yet to compute Fndr and other FDR quantities a suitable value for *y*_*c *_must be specified. Fortunately, for most data sets the location of the cutoff point *y*_*c *_need only be known approximately. The "FNDR" strategy employed in "fdrtool" proceeds in two stages. In the first step, the mixture model is fit approximately, which leads to an approximate Fndr curve from which an approximately optimal *y*_*c *_is obtained. In the second step truncated maximum-likelihood estimation on the basis of the approximate cutoff *y*_*c *_is used for a refined fit of the mixture model which in turn allows to compute FDR quantities of interest.

**Table 3:** Various choices of normal truncation points implemented in "locfdr".

*Version*	*Released*	*Truncation Point*	*Reference*
1.1–1	July 2006	*z*_0 _= 2	
1.1–3	December 2006	$z_{0}=\hat{\mu }+b\hat{\sigma }$ with *b *= 3.55 – 0.44 log_10_(*m*), $\hat{\mu }$ = median(*z*_*i*_) and $\hat{\sigma }$ = IQR(*z*_*i*_)/1.349.	
1.1–6	November 2007	as version 1.1–3, but with *b *= max(1, 4.3*m*^-0.112966^)	[28]

Note that $\hat{\mu }$ and $\hat{\sigma }$ are robust location and scale estimates, respectively.

A simple approximate fit of the null model is achieved by matching its median $F_{0}^{-1}$ (1/2; *θ*) with that of the observed *y*_*i*_. Thus, a robust estimate of scale is used just as in the "locfdr" algorithm, see Table 3 (note that the median for the half-normal distribution corresponds to the interquartile range (IQR) of the corresponding normal with mean zero). Subsequently, after converting the test statistics into *p*-values an approximate estimate of the null proportion is determined by estimating *η*_0 _for various cutoff-points and finally settling for the 0.1 quantile of the resulting distribution of corresponding *η*_0_.

In addition to selecting *y*_*c *_by the above "FNDR" approach, further methods available in the "fdrtool" package include the "locfdr" cutoff method [28] and the specification of the fraction of data points to be considered for estimating the empirical null. In a practical analysis it is always advisable to conduct the FDR calculations for various choices of *y*_*c*_, (even though truncated maximum-likelihood appears to be fairly robust with regard to *y*_*c*_).

#### Gluing it all together

With the above preliminaries, a general algorithm for estimating Fdr and fdr from an arbitrary test statistics *y*_*i *_can be put together as follows:

1. Determine a suitable truncation point *y*_*c*_.

2. Estimate the null model and its parameters, yielding ${\hat{\eta }}_{0}$ and $\hat{\theta }$.

3. Convert test statistics into *p*-values via *p*_*i *_= 1 - *F*_0_(*y*|$\hat{\theta }$).

4. Estimate the PDF ${\hat{f}}_{p}$(*p*) and CDF ${\hat{F}}_{p}$(*p*) of the *p*-values using the modified Grenander estimator (note that this requires ${\hat{\eta }}_{0}$).

5. Compute estimates of Fdr and fdr values based on *p*-values:

$$\begin{array}{c} {\hat{\text{fdr}}}_{p}(p)=\frac{{\hat{\eta }}_{0}}{{\hat{f}}_{p}(p)}\\ {\hat{\text{Fdr}}}_{p}(p)=\frac{{\hat{\eta }}_{0}p}{{\hat{F}}_{p}(p)} \end{array}$$

6. Compute estimated Fdr and fdr values as a function of the original test statistics *y*:

$$\begin{array}{c} \hat{\text{fdr}}(y)={\hat{\text{fdr}}}_{p}(1-{\hat{F}}_{0}(y))\\ \hat{\text{Fdr}}(y)={\hat{\text{Fdr}}}_{p}(1-{\hat{F}}_{0}(y)) \end{array}$$

7. Compute CDF and PDF on the *y*-scale:

$$\begin{array}{c} \hat{f}(y)={\hat{\eta }}_{0}\frac{{\hat{f}}_{0}(y)}{\hat{\text{fdr}}(y)}\\ \hat{F}(y)=1-{\hat{\eta }}_{0}\frac{1-{\hat{F}}_{0}(y)}{\hat{\text{Fdr}}(y)} \end{array}$$

Note that this transformation is directly derived from the definition of fdr and Fdr in Eqs. 6 and 7.

8. Estimate alternative sub-density:

$$\begin{array}{c} {\hat{F}}_{A}(y)=\frac{\hat{F}(y)-{\hat{\eta }}_{0}{\hat{F}}_{0}(y)}{1-{\hat{\eta }}_{0}}\\ {\hat{f}}_{A}(y)=\frac{\hat{f}(y)-{\hat{\eta }}_{0}{\hat{f}}_{0}(y)}{1-{\hat{\eta }}_{0}} \end{array}$$

## Results and discussion

### Computer simulations for *p*-value-based analyses

In order to assess the performance of the "fdrtool" algorithm it was compared with a number of other FDR procedures. Specifically, the R packages "fdrtool" version 1.2.4, "qvalue" version 1.1 [7], "locfdr" version 1.1–6 [4], "twilight" version 1.14.1 [30], "kerfdr" version 1.0.0 [23] and "nFDR" version 0.0 [24] were investigated.

First, FDR approaches based on *p*-values were studied. As generative model *p*-values were simulated from a mixture of the uniform *U*(0, 1) with either the truncated exponential density $f_{A}(p;a)=\frac{a}{\exp(a)-1}\exp(a(1-p))$ or with the uniform *f*_*A*_(*p*; *a*) = *U *(0, *a*). Sample size and mixture model parameter *a *were varied, and from each generated data set the proportion of null *p*-values, and the squared error of the local FDR and the tail area-based FDR was estimated. The references for computing the squared error were the theoretical Fdr and fdr values derived from the assumed model.

**Figure 5:**
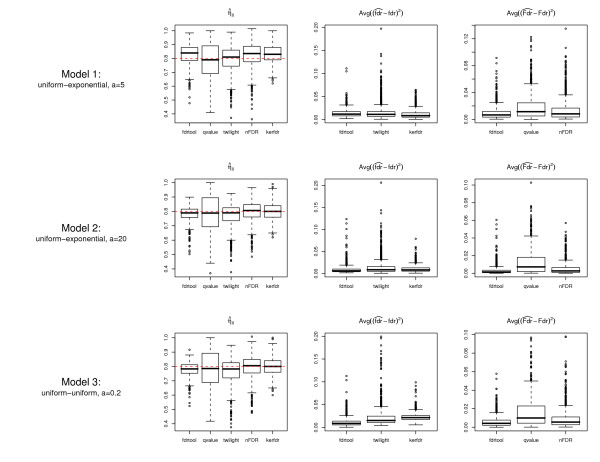
**Comparison of the estimates of the proportion of null *p*-values and the squared error of Fdr and fdr for various *p*-value-based FDR estimation procedures under three different simulation scenarios.** The box plots summarize the estimates from *B *= 1000 repetitions of the simulations. The sample size (i.e. the number of multiple tests) was *m *= 200.

Fig. 5 displays the results for three different cases: "model 1" is a uniform-exponential mixture with *η*_0 _= 0.8 and *a *= 5, "model 2" is identical to "model 1" except for *a *= 20, and "model 3" utilizes the uniform-uniform mixture with *a *= 0.2. In all cases there were *B *= 1000 repeats and the number of *p*-values was *m *= 200.

The first column of Fig. 5 shows the accuracy in estimating *η*_0_. Overall, the "kerfdr" and the "fdrtool" algorithms exhibit the smallest variability at the expense of a slightly biased estimate of *η*_0_, especially for "model 1". In contrast, "qvalue" always produces nearly unbiased estimates but has a much higher variance. The "twilight" and "nFDR" are similar to "qvalue", but are less variable.

The second and third columns of Fig. 5 depict the error in the actually estimated Fdr and fdr values for various algorithms under the three model scenarios. In terms of correctly estimating fdr values all investigated packages with capability of computing local FDR (i.e. "fdrtool", "kerfdr", and "twilight") perform roughly equally well across all scenarios For "model 3" the "fdrtool" appears to have a slight advantage over the competing approaches. When comparing the accuracy of Fdr values "fdrtool" outperforms both "qvalue" and "nFDR", even though the differences are not large. The squared error of Fdr computed by "qvalue" and by "nFDR" exhibits more extreme outliers than those of "fdrtool".

### Simulations and analysis of gene expression data for *z*-scores

In a further simulation study estimation of FDR from *z*-scores was considered with empirical null modeling. Specifically, data were simulated from a mixture of the normal distribution *N*(*μ *= 0, *σ *= 2) with the symmetric uniform alternatives *U*(-10, -5) and *U*(5, 10).

**Figure 6:**
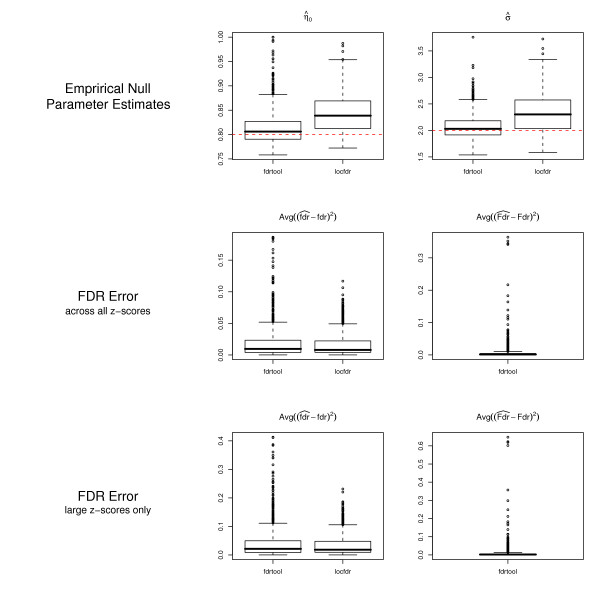
**Accuracy and variability of estimates of the null proportion *η*_0 _and the scale parameter *s *as well as the squared error of Fdr and fdr for *z*-score based algorithms.** As in Fig. 5 *m *= 200 and *B *= 1000 was used. For the plots in the third row only *z*-scores with |*z*| > 2 were used.

An example of the results from the simulations for sample size *m *= 200, *B *= 1000 repeats and *η*_0 _= 0.8 is shown in Fig. 6. The estimates of the mixing parameter *η*_0 _and of the scale parameter *s *are slightly upwardly biased in "locfdr" but more importantly they also exhibit larger variability compared to "fdrtool". The mean squared error of the fdr vales are similar for both algorithms. Note that in this simulation a 75% quantile cutoff point was employed in "fdrtool" for the truncated maximum-likelihood estimation of the null model. The third row of Fig. 6 shows the FDR errors for *z*-scores with absolute values larger than 2. In this domain both investigated algorithms perform again very similar, but the average error is larger in comparison to the situation when all *z*-scores are included in the analysis. In order to further compare the empirical null modeling a HIV and a breast cancer (BRCA) microarray gene expression data set was reanalyzed, following [31] and [4]. For the detailed biological background and the experimental setup it is referred to the original papers.

**Figure 7:**
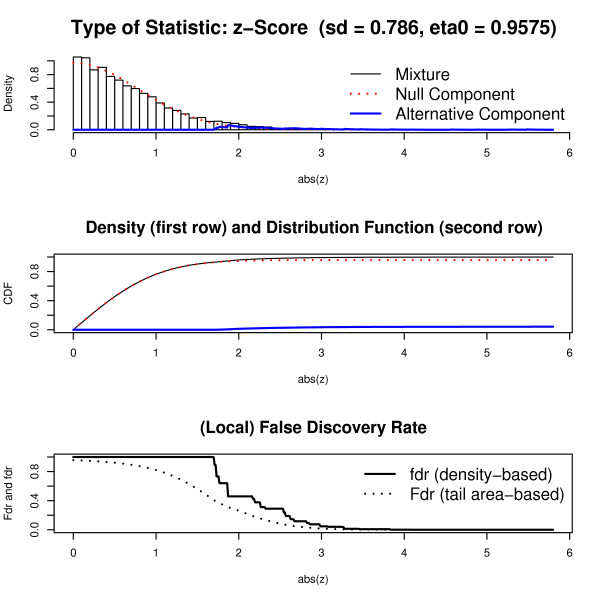
**Graphical output provided by "fdrtool" for the HIV data set.** The first row shows the densities, the second the distribution function and the last row the local and tail area-based false discovery rates.

The HIV data consists of 7680 *z*-scores. The fit of "fdrtool" to the median-centered data is shown in Fig. 7. Specifically, the standard deviation of the null normal density was estimated to be $\hat{\sigma }$ = 0.786 and the mixing parameter ${\hat{\eta }}_{0}$ = 0.9575. The number of discoveries with a fdr value smaller than 0.2 was 119. The "locfdr" algorithm finds a very similar null model, namely $\hat{\sigma }$ = 0.754 and ${\hat{\eta }}_{0}$ = 0.9342. Corresponding to the smaller ${\hat{\eta }}_{0}$ "locfdr" detects 160 significant *z*-scores with fdr < 0.2.

The breast cancer data has size 3226. After median-centering the data were again supplied to both the "fdrtool" and "locfdr" packages. Both algorithms indicated that there was overdispersion ($\hat{\sigma }$ = 1.51 versus $\hat{\sigma }$ = 1.55) and and the proportion of null values was estimated to be ${\hat{\eta }}_{0}$ = 1. Correspondingly, for the BRCA data there were no significant *z*-scores (note that this is in contrast to claims otherwise in [31]). In short, "fdrtool" and "locfdr" provide very similar analyzes both in terms of empirical null estimation and inferred fdr values.

### Computational efficiency

Finally, the investigated FDR procedures were also compared in terms of computational efficiency. The (by a large margin) slowest program is "twilight". In contrast, the fastest algorithms are "fdrtool", "locfdr" and "qvalue", followed by "kerfdr" and "nFDR".

## Conclusion

False discovery rate analysis is a key statistical innovation that has found widespread application in the study of high-dimensional data. One of the intriguing aspects of FDR is that can be understood both from a frequentist and Bayesian perspective. This has lead to a plethora of FDR criteria and FDR inference procedures.

The goal for the development of the "fdrtool" procedure was to establish a common framework that brings together the most compelling features of existing FDR methods. Specifically, novel features of the proposed "fdrtool" algorithm include

• a unified treatment of *p*-values and other test statistics, with identical algorithms and learning procedures,

• simultaneous and coherent estimation of both Fdr and fdr,

• empirical null modeling for test statistics other than *z*-scores,

• a method for selecting the truncation point based on controlling FNDR, and

• a simple semiparametric model using a modified Grenander density estimator.

Hence, "fdrtool" allows to compute local FDR values from *p*-values but also Fdr values from *z*-scores while taking account of an empirical null model. Despite the generality of the algorithm, it was shown that the accuracy of the algorithm is on par with the best competing yet more specialized FDR procedures. Moreover, the modular structure of the "fdrtool" procedure facilitates future extensions.

In summary, the "fdrtool" package and algorithm constitutes a comprehensive and feature-rich tool for a wide range of FDR-type analyzes.

During revision a referee pointed out that the distribution of observed *p*-values might be *U*-shaped [20]. This occurs, among other possibilities, if the null model is misspecified. As a result, the computed null *p*-values do not follow a uniform distribution, and thus by definition are improper. "fdrtool" cannot be applied directly to improper *p*-values, however, in these instances it might instead be preferable to conduct the FDR analysis on the level of the original test statistics.
